# Noninvasive sampling of the small intestinal chyme for microbiome, metabolome and antimicrobial resistance genes in dogs, a proof of concept

**DOI:** 10.1186/s42523-023-00286-0

**Published:** 2023-12-16

**Authors:** Julie Menard, Sahar Bagheri, Sharanya Menon, Y. Tina Yu, Laura B. Goodman

**Affiliations:** 1 Department of Veterinary Diagnostic and Clinical Sciences, Faculty of Veterinary Medicine, University of Calgary, Calgary, AB Canada; 2 International Microbiome Center, Snyder Institute for Chronic Diseases, Cummings School of Medicine, University of Calgary, Calgary, AB Canada; 3 Nimble Science Ltd., Calgary, AB Canada; 4 Baker Institute for Animal Health and Department of Public and Ecosystem Health, College of Veterinary Medicine, Cornell University, Ithaca, NY USA

**Keywords:** Microbiome, Metabolome, Gastrointestinal tract, Capsule, Antimicrobial resistance genes

## Abstract

**Background:**

The gastrointestinal microbiome and metabolome vary greatly throughout the different segments of the gastrointestinal tract, however current knowledge of gastrointestinal microbiome and metabolome in health and disease is limited to fecal samples due to ease of sampling. The engineered Small Intestinal MicroBiome Aspiration (SIMBA™) capsule allows specific sampling of the small intestine in humans. We aimed to determine whether administration of SIMBA™ capsules to healthy beagle dogs could reliably and safely sample the small intestinal microbiome and metabolome when compared to their fecal microbiome and metabolome.

**Results:**

Eleven beagle dogs were used for the study. Median transit time of capsules was 29.93 h (range: 23.83–77.88). Alpha diversity, as measured by the Simpson diversity, was significantly different (*P* = 0.048). Shannon diversity was not different (*P* = 0.114). Beta diversity results showed a significant difference between capsule and fecal samples regarding Bray–Curtis, weighted and unweighted unifrac (*P* = 0.002) and ANOSIM distance metric s (R = 0.59, *P* = 0.002). In addition to observing a statistically significant difference in the microbial composition of capsules and feces, distinct variation in the metabolite profiles was seen between the sample types. Heat map analysis showed 16 compounds that were significantly different between the 2 sampling modes (adj-*P* value ranged between 0.004 and 0.036) with 10 metabolites more abundant in the capsule than in the feces and 6 metabolites more abundant in the feces compared to the capsules.

**Conclusions:**

The engineered Small Intestinal MicroBiome Aspiration (SIMBA™) capsule was easy and safe to administer to dogs. Microbiome and metabolome analysis from the capsule samples were significantly different than that of the fecal samples and were like previously published small intestinal microbiome and metabolome composition.

**Graphical abstract:**

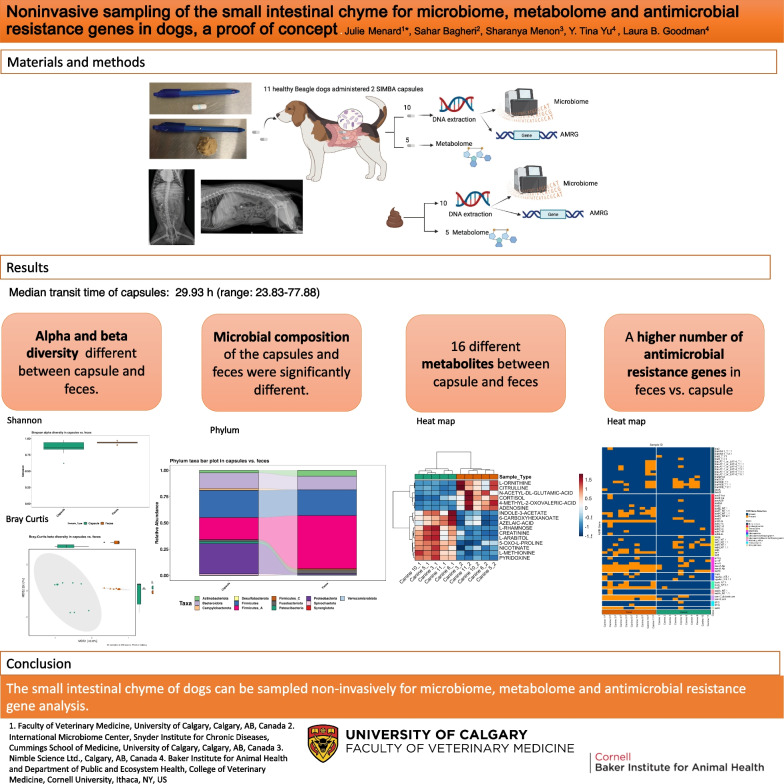

**Supplementary Information:**

The online version contains supplementary material available at 10.1186/s42523-023-00286-0.

## Background

The gastrointestinal microbiome and metabolome are impaired in several disease processes in both humans and dogs. In humans, gastrointestinal (GI) disease processes such as *Clostridioides difficile* infection [1–5] and numerous non -GI disease such as asthma, [6] epilepsy, [7] arthritis [8], autism [9], cardiovascular disease [10], and renal disease [11] have documented changes in microbiome and metabolome compared to healthy humans. Similarly, changes in fecal microbiome and metabolome have been seen in dogs suffering from GI disease such as exocrine pancreatic insufficiency (EPI) [12], inflammatory bowel disease (IBD) [3], acute [13], and chronic diarrhea in dogs [14] as well as obesity [15]. These changes in microbiome and metabolome within disease processes highlight possible underlying mechanistic processes and possible future treatment avenues for therapies. However, the composition of the microbiota and metabolome is not uniform throughout the GI tract (GIT) [5, 16] The effect of digestion, microbial fermentation or other enzymatic action produces significant differences in composition and abundance of bacterial classes and a wide array of nutrients and metabolites. Due to ease of sampling, most of the studies cited above rely on fecal microbiome sampling, which differs in composition from the stomach, duodenum, jejunal, ileal and colon microbiome [17]. In dogs, sampling of other segments of the gastrointestinal tract relies on invasive techniques such as endoscopic biopsies performed under general anesthesia [18, 19], creation of a surgical jejunal fistula [20] or postmortem collection of intestinal chyme following euthanasia [16]. Engineered capsules recording pH and pressure [21] or used for video endoscopy [22, 23] have been previously used in both healthy and sick dogs. The engineered Small Intestinal MicroBiome Aspiration (SIMBA™) capsule allows specific sampling of the small intestine. The capsule has a shell with a gastric-resistant coating and it dissolves passively from the intestinal chyme once the coating is exposed in a neutral pH. Studies in humans have shown significant difference in microbial and metabolome composition between the capsule sample and fecal samples [24–26]. Non-invasive sampling of the small intestinal chyme coupled with paired fecal microbiome and metabolomic analysis could provide valuable insights in disease processes such as IBD, EPI and others. We aimed to determine whether administration of SIMBA™ capsule to healthy dogs could reliably and safely sample the small intestinal microbiome and metabolome compared to fecal microbiome and metabolome. In addition, we aimed to determine the mean transit time for SIMBA capsules in beagles. Lastly, we aimed to compare recovery of antimicrobial resistance genes from the small intestinal chyme and feces. We hypothesize that SIMBA capsules would be safe and easy to administer and recover in dogs. In addition, we hypothesize that microbiome, metabolome, and recovery of AMR genes would differ between the capsule and the feces.

## Results

Eleven beagle dogs used for clinical skills teaching at a veterinary school were used for the study. Of those, 8 were female spayed and 3 were castrated male. Mean body weight was 10.09 kg (Std ± 1.5). All dogs fasted overnight and ate two meatball each containing a capsule in the next morning. Only one dog was seen chewing both meatballs.

### Transit times

**Fig. 1:**
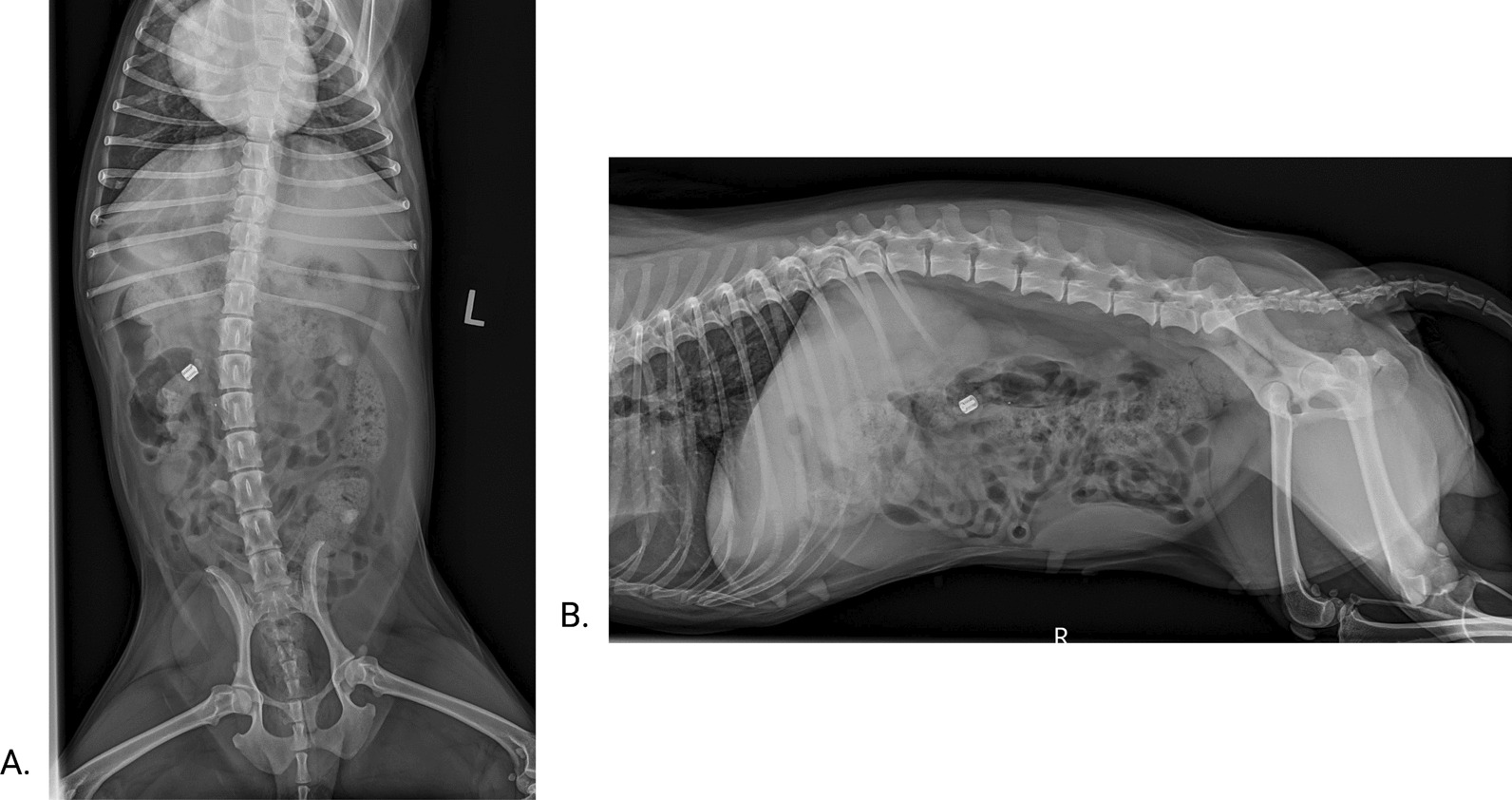
**A.** Ventro-dorsal abdominal radiograph and **B.** Right lateral abdominal radiograph at 73 h post capsule ingestion. The radio-opaque capsule is visualized within the ascending colon

Median transit time of engineered capsules was 29.93 h (range: 23.83–77.88). Nine dogs defecated the capsules within the same bowel movement. In one of the dogs, where both capsules were not passed simultaneously, an adverse effect was seen, with the dog vocalizing and inspecting its rear end. Physical examination revealed a single capsule in the rectum without any feces. The capsule was manually retrieved with gentle digital palpation and lubricant (three hours after the first capsule). No other adverse events were recorded. The second dog which didn’t defecate both capsules within the same bowel movement had a slower transit time. Physical examination in the dog with the missing capsule was unremarkable. Two view abdominal radiographs performed at the 72 h mark identified the capsule in the ascending colon (Fig. 1). The dog continued with normal activity and feeding schedule. The capsule was defecated 77.88 h post administration (6 h after the first capsule) without complications.

### Capsule samples

Median weight of the sample collected by the capsule was 73.60 mg IQR [47.8–88.53] range (12.6–108.8). The Grubb’s test (G = 2.04207 *P* = 0.0974) failed to identify any outliers. Median of mean sample weight 69.42 mg IQR [58.39–80.05] retrieved by the capsule was not correlated to the median of the mean transit time 29.89 h IQR [27.85–30.30] (r = 0.2727 *P* = 0.448, 95% CI − 0.4308898; 0.7701127).

### DNA extraction from capsules

DNA was extracted from SIMBA capsule and fecal samples using the Qiagen QIAamp PowerFecal Pro DNA Kit. DNA was extracted from the heaviest capsules for each dog. During the DNA extraction step, 2 capsules samples were impacted by process-related sample loss and no DNA was extracted from either sample. As such, they were excluded from downstream analysis. Additionally, 3 capsule samples DNA may have been impacted by process-related sample contamination. To supplement the loss of 2 capsule samples and control for potential contamination in 3 capsule samples, the second out of two capsule samples collected from the respective dog also underwent DNA extraction and were included in the downstream 16S sequencing analysis. The heaviest capsule was submitted for DNA analysis in 6 dogs. In 5 dogs, DNA was extracted from both capsules and a total of 14 DNA capsule samples submitted for initial downstream 16S sequencing analysis. One dog had a larger bowel movement when defecating the capsules. Each capsule was collected and stored with the adjoining feces. Hence 12 fecal samples had DNA extracted and submitted for initial 16S sequencing. The 16S rRNA amplicon sequencing with V4 region targeting primers was performed on all submitted samples (14 capsules and 12 feces) prior to final analysis. The post-PCR amplification quality check performed on a 1% agarose gel demonstrated that 9/14 capsule samples and 12/12 fecal samples amplified sufficiently to produce a positive band on the gel. Following bioinformatic analysis, one capsule had low number of reads (this capsule was chewed on) and was excluded from final analysis. Additionally, one capsule sample was an outlier and clustered with feces analysis. Given the fecal-like microbiome profile of the sample, it was excluded from final downstream analysis as well. Visual analysis was performed in the 3 paired capsules samples for which potential contamination was suspected during DNA extraction. Ultimately, 10 paired capsule and fecal samples DNA analysis were included in the final 16S sequencing and AMR genes analysis regardless of the post PCR amplification quality check step and are presented below. All remaining 6 capsule samples and their corresponding fecal samples were processed for metabolomic analysis (Table 1). The mean DNA concentration from capsules was 0.03 ng/uL ± 0.02 and in the feces 6.59 ng/uL ± 0.70 1 5 which was significantly different (paired *t* test *p* = 2.817e−07).

**Table 1:** This table contains data pertaining to each canine enrolled in the study with corresponding details of capsule and paired fecal samples collected from each canine

Canine				Capsule sample						Paired feces samples		
Canine#	Body Weight (kg)	Sex	Diet	Capsule #	Internal Transit Time (hr:mm)	Sample Weight (mg)	DNA Conc. (ng/ul)	Analysis Method	Sample Included in final Analysis? (Y/N); Reason	DNA Conc. (ng/ul)	Analysis method	Sample Included in final Analysis? (Y/N); Reason
1	8.5	FS	Royal Canin Hypoallergenic Hydrolyzed protein	1	29:93	94.1	OOR	16S sequencing AMRG	Y	5.58	16S sequencing AMRG	Y
				2	29:93	18.2		Metabolome	N; Low post-dilution volume		Metabolome	N; Paired capsule sample removed from analysis
2	13.0	MC	Royal Canin Hypoallergenic Hydrolyzed protein	1	30:28	105.8	OOR	16S sequencing AMRG	Y; Possible contamination (not seem on 16S analysis)	7.92	16S sequencing AMRG	Y
				2	30:28	75.2	OOR	16S sequencing AMRG	N; higher weight capsule was used in final analysis			
3	9.6	FS	Royal Canin Dental	1	47:30	71.0		Metabolome	Y		Metabolome	Y
				2	47:30	74.4	0.011	16S sequencing AMRG	Y	6.06	16S sequencing AMRG	Y
4	10.4	FS	Purina Veterinary Diets Essential Care Dry + Science Diet Beef & Barley Entree Canned	1	30:30	15.0	OOR	16S sequencing AMRG	N: higher weight capsule was used in final analysis	7.14	16S sequencing AMRG	Y
				2	30:30	49.0	0.037	16S sequencing AMRG	Y; Possible contamination (not seem on 16S analysis)			
5	11.5	MC	Royal Canin Dental	1	27:82	72.4	0.0682	16S sequencing AMRG	Y	7.4	16S sequencing AMRG	Y
				2	27:82	66.0		Metabolome	Y		Metabolome	Y
6	11.4	FS	Royal Canin Dental	1	23:83	42.9		Metabolome	Y		Metabolome	Y
				2	23:83	87.3	0.0102	16S sequencing AMRG	Y	6.92	16S sequencing AMRG	Y
7	8.2	FS	Royal Canin Satiety	1	47:33	108.8	0.0334	16S sequencing AMRG	Y	5.68	16S sequencing AMRG	Y
				2	47:33	77.6			N; Loss of sample			
8*	11.0	MC	Royal Canin Dental	1	29:85	12.6	OOR	16S sequencing AMRG	Y	7.14	16S sequencing AMRG	Y
				2	29:85	17.6	OOR	16S sequencing AMRG	N; Possible contamination and low number of reads	7.24	16S sequencing AMRG	N
9^	10.0	FS	Royal Canin Dental	1	26:40	92.2	OOR	16S sequencing AMRG	N; Removed from analysis due to clustering with fecal sample	7.66	16S sequencing AMRG	N; Paired capsule sample removed from analysis
				2	29:48	72.8		16S sequencing AMRG	N; Loss of sample		16S sequencing AMRG	N; Paired capsule sample lost
10#	8.8	FS	Royal Canin Dental	1	71:38	78.6		Metabolome	Y		Metabolome	Y
				2	77:88	93.1	0.0332	16S sequencing AMRG	Y	5.84	16S sequencing AMRG	Y
11	8.6	FS	Royal Canin Dental	1	26:37	61.8		Metabolome	Y		Metabolome	Y
				2	26:37	77.5	0.0114	16S sequencing AMRG	Y	7.08	16S sequencing AMRG	Y

Each canine was randomly designated a number at the beginning of the study for sample and analysis tracking. The sex of each dog is listed as either Female-Spayed (FS) or Male-Castrated (MC). Data pertaining to capsule and paired fecal samples include the internal transit time in hours of the capsule which tracked the time from capsule ingestion to excretion. The capsule sample weight refers to the weight of intestinal chyme (in mg) collected within the capsule. The DNA concentration in both capsule and fecal samples are recorded in ng/ml, where “OOR” refers to an Out of Range–too low (< 0.0100 ng/ul) reading on the Qubit 4 Fluorometer used to quantify the concentration. The analysis method that each capsule and paired fecal samples were designated to is described as either 16S (16S rRNA Sequencing) and antimicrobial resistance gene (AMRG) or Metabolome (untargeted HILIC Liquid Chromatography—Mass Spectrometry metabolomics). *Was seen chewing on both meatballs. ^capsule 1 was found alone in the rectum and was manually removed. #Hadn’t defecated the 2nd capsule by the 72 h mark and had 2 view abdominal radiographs taken (Fig. 1)

### Microbiome analysis

**Fig. 2:**
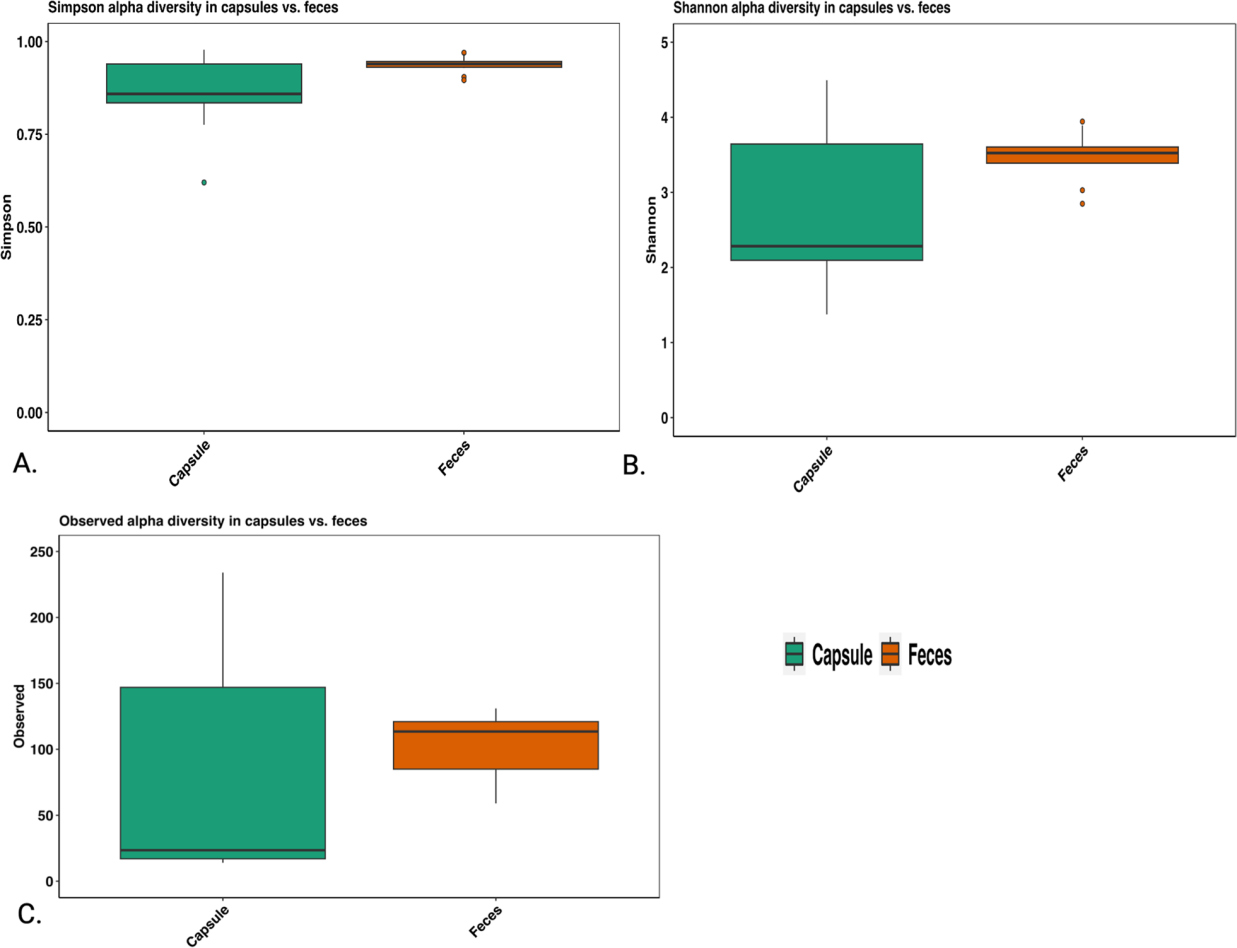
**A.** Alpha diversity Simpson index (Wilcoxon signed rank exact test *P* = 0.048) **B.** Shannon alpha diversity (paired *t* test *P* = 0.1136) **C.** Observed diversity (paired *t* test *P* = 0.028)

**Fig. 3:**
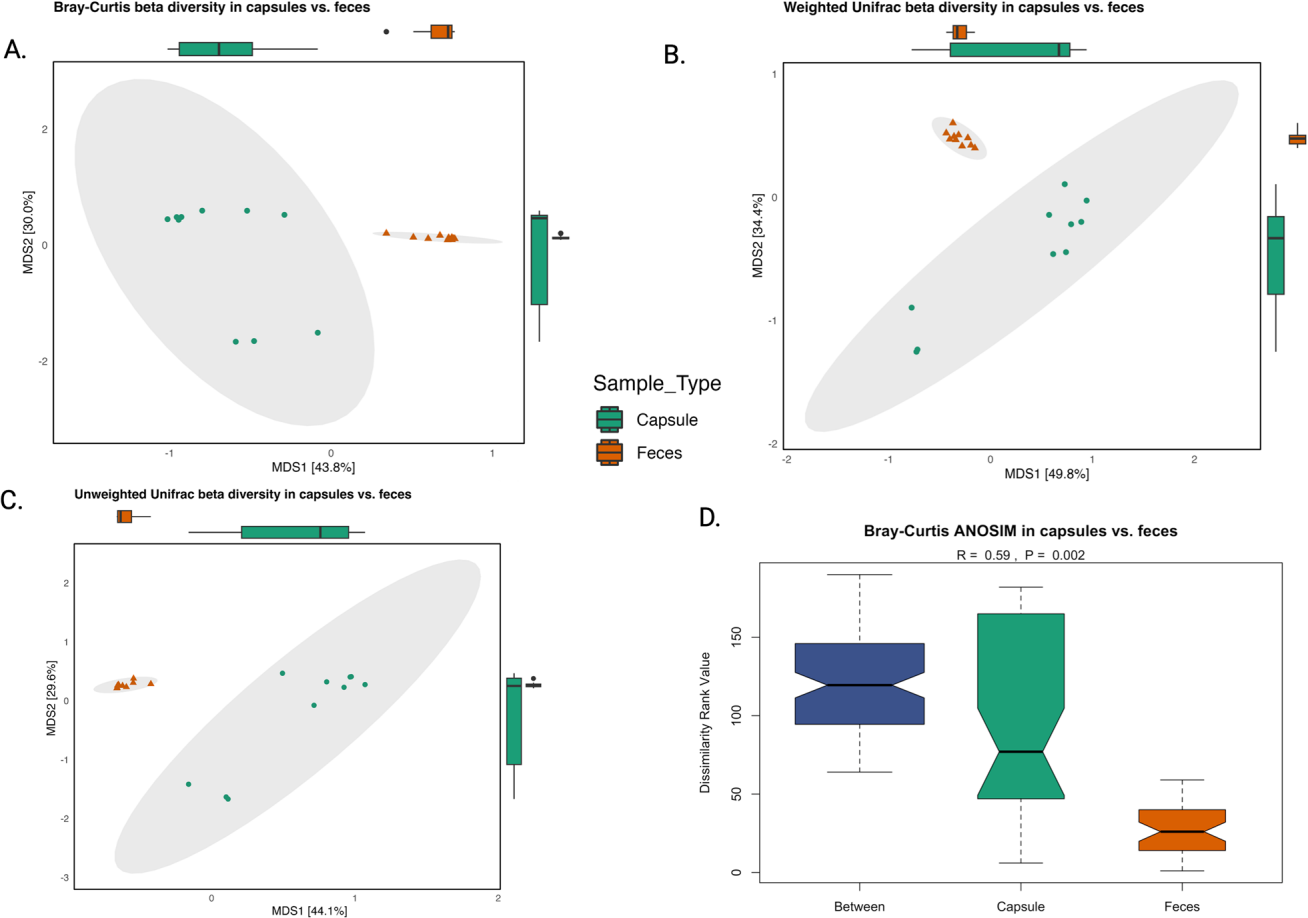
Beta diversity significantly differed between capsule and feces as shown by: **A.** Bray Curtis distance (*P* = 0.002) **B.** Weighted unifrac distance (*P* = 0.002) **C.** Unweighted unifrac distance (*P* = 0.002) D. ANOSIM Bray Curtis distance metrices (*P* = 0.002)

Alpha diversity, as measured by the Simpson diversity (Fig. 2A) and observed diversity indexes (Fig. 2C) differed significantly between the capsule and feces (*P* = 0.048 and *p* = 0.028 respectively). However, Shannon diversity (Fig. 2B) did not differ between the two sample types (*P* = 0.114). Beta diversity results also showed a significant difference between capsule and fecal samples regarding Bray–Curtis, weighted and unweighted unifrac (*P* = 0.002 respectively) and ANOSIM distance metrices (R = 0.59, *P* = 0.002). (Fig. 3).

**Fig. 4:**
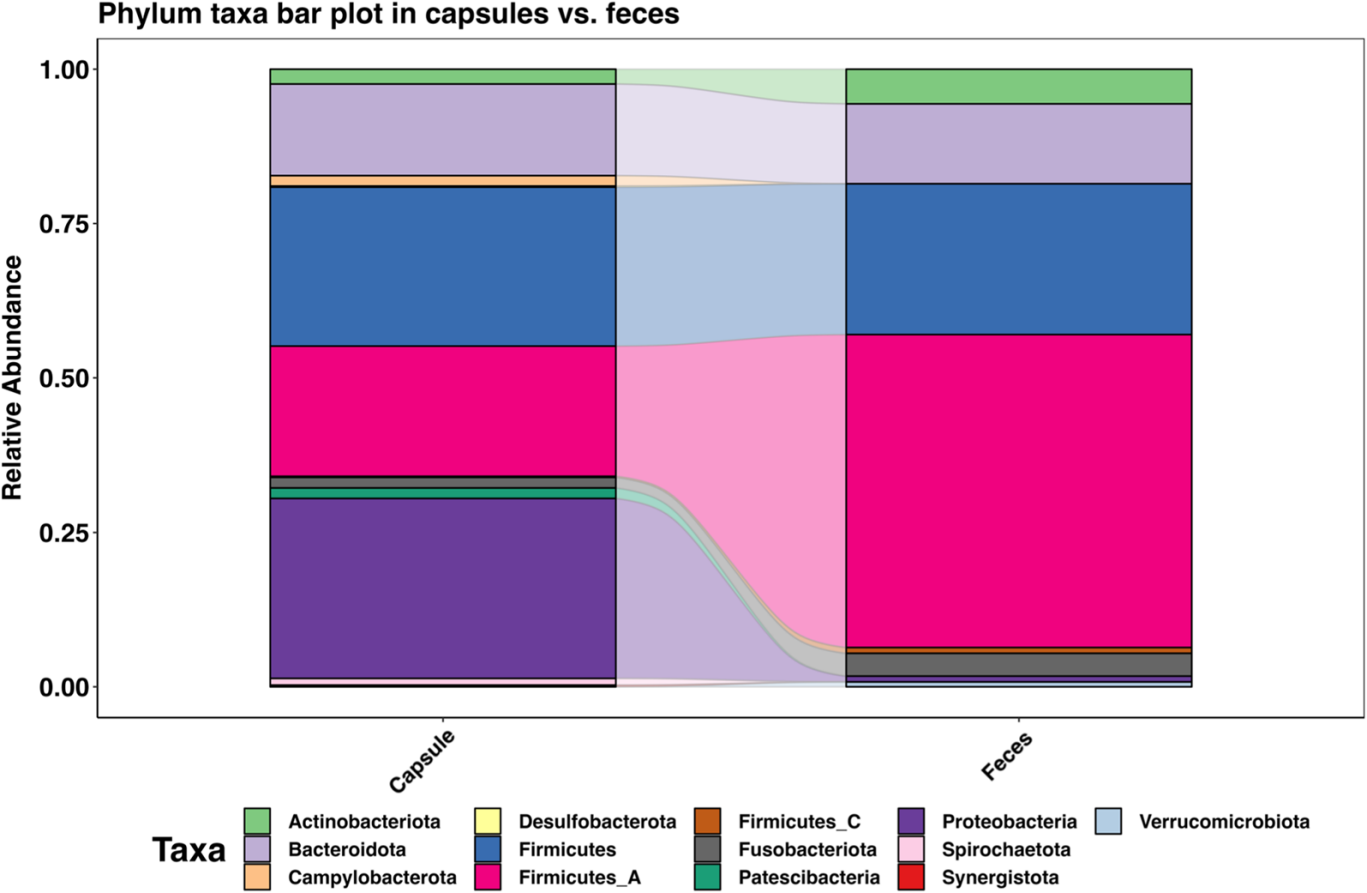
**A.** Phylum taxa plot of capsules and fecal samples. **B.** Venn diagram comparison between capsule and fecal samples at ASV level

**Fig. 5:**
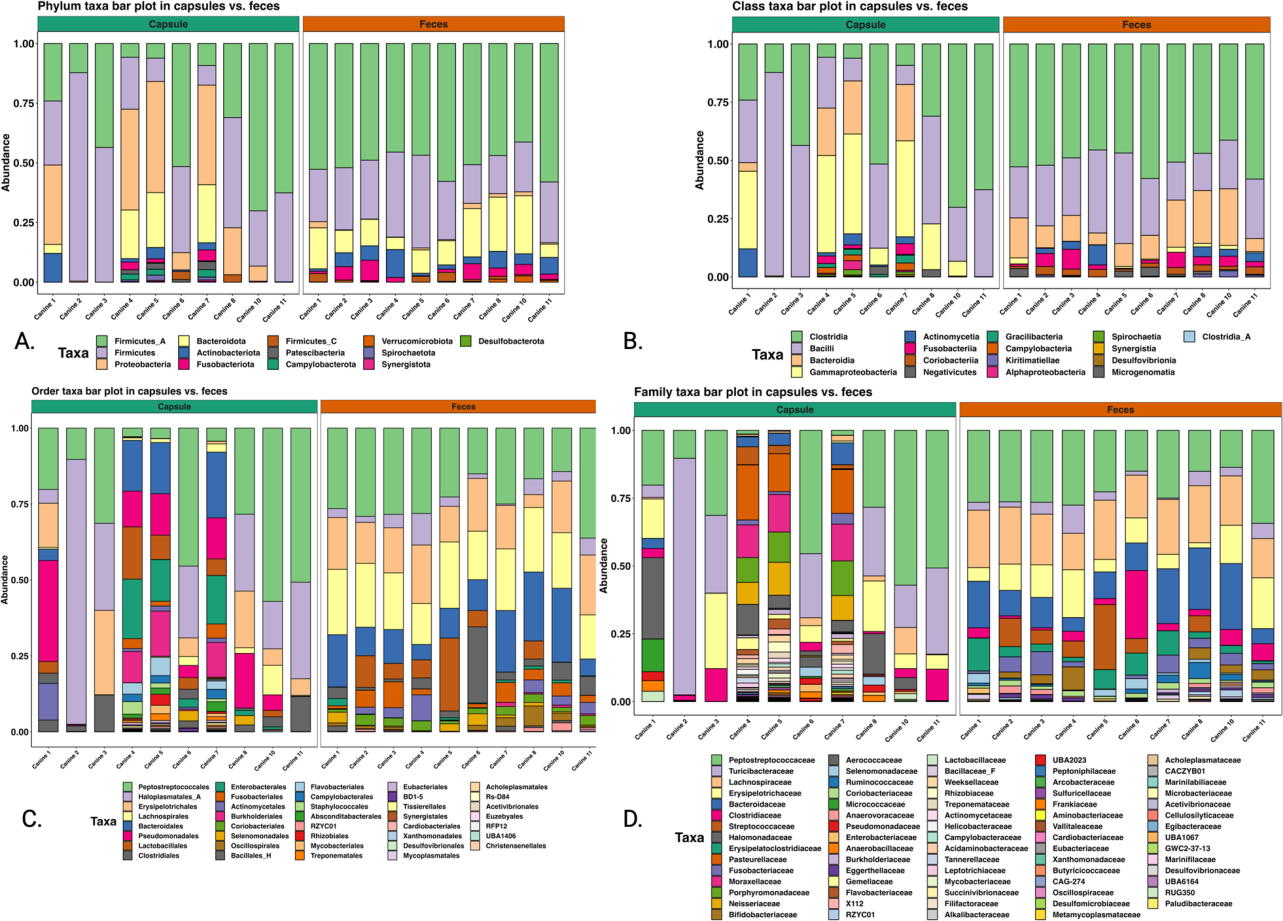
**A.** Phylum **B.** Class **C.** Order and **D.** Family of bacterial composition of the capsule sample (right-hand side) and the fecal sample (left-hand side)

**Fig. 6:**
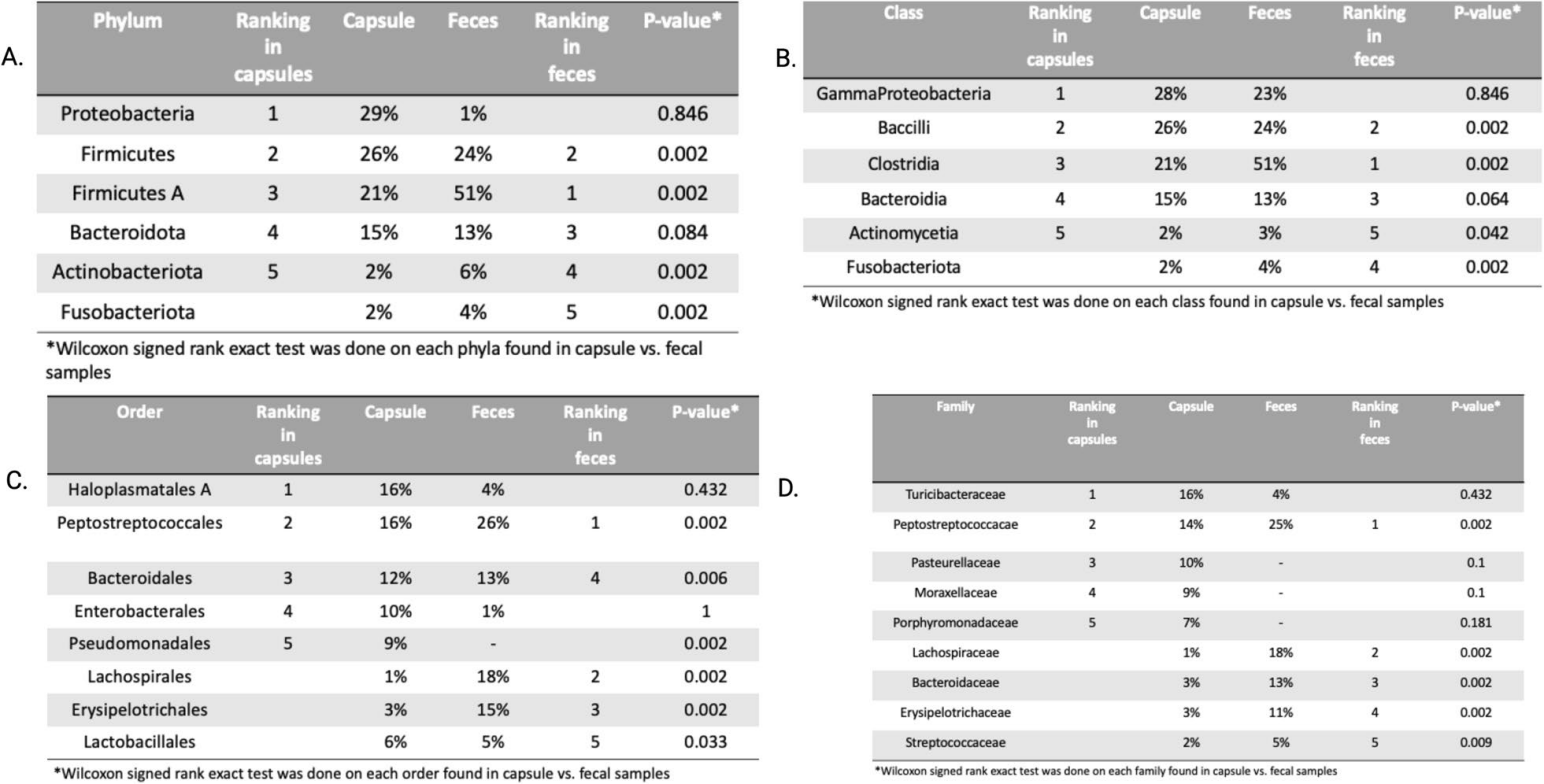
Top 5 of each **A.** Phylum, **B.** Class, **C.** Order, and **D.** Family in each sample with Wilcoxon signed rank exact test each bacterial classification between the capsules and the feces

**Fig. 7:**
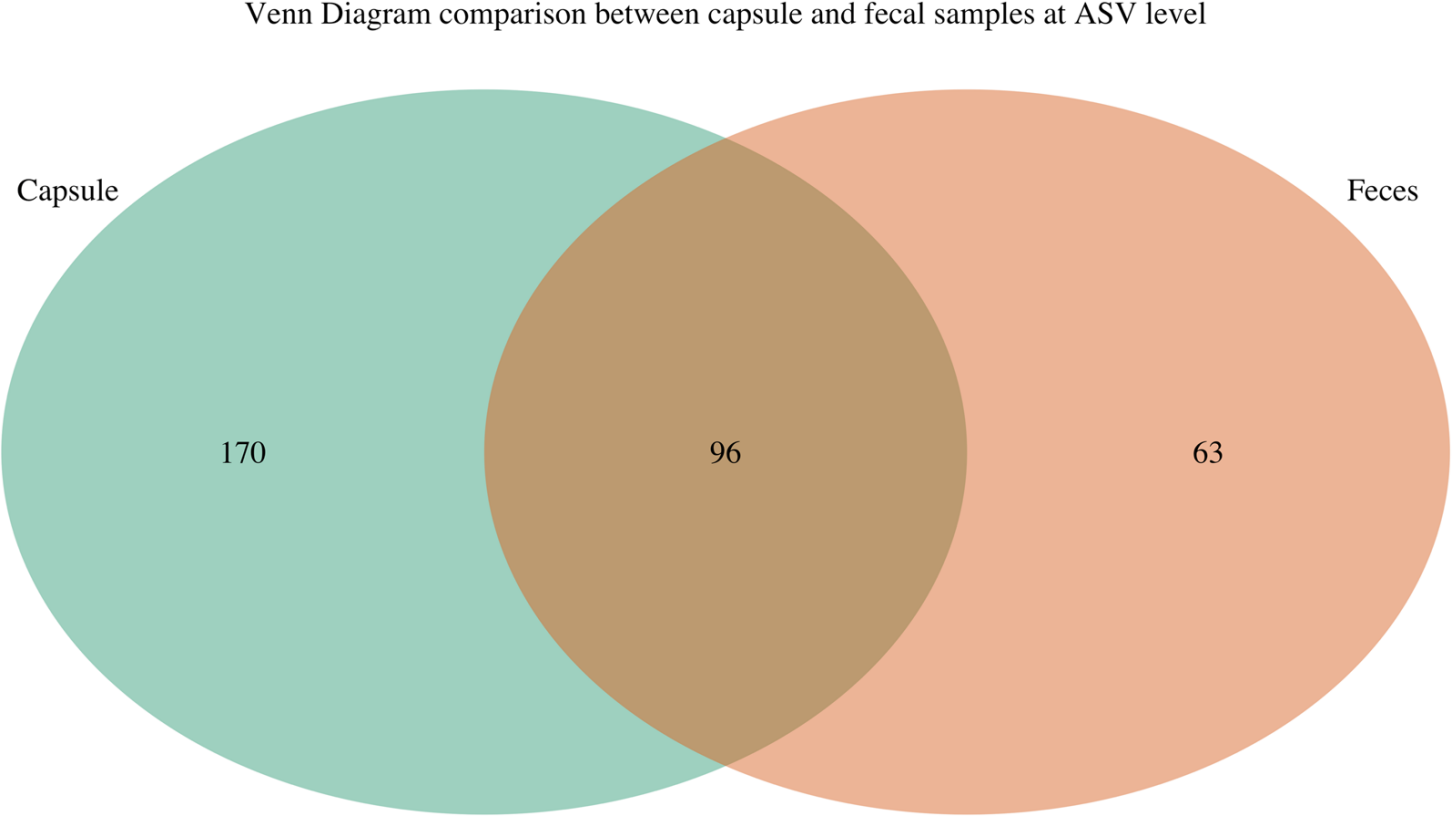
Venn diagram comparison between capsule and fecal samples at ASV level

The composition of capsule and fecal microbiome differed significantly as shown in Figs. 4, 5, 6 and 7. The capsule sample exhibited a coexistence of both aerobic bacteria, (*Bacilli*, *Gamma proteobacteria*, and *Actinomyceta*) and anaerobic classes (Proteobacteria), whereas the fecal sample was predominantly populated by anaerobic bacteria (*Clostridia*, *Bacteroidetes*, and *Fusobacterium*).

### Metabolome analysis

**Fig. 8:**
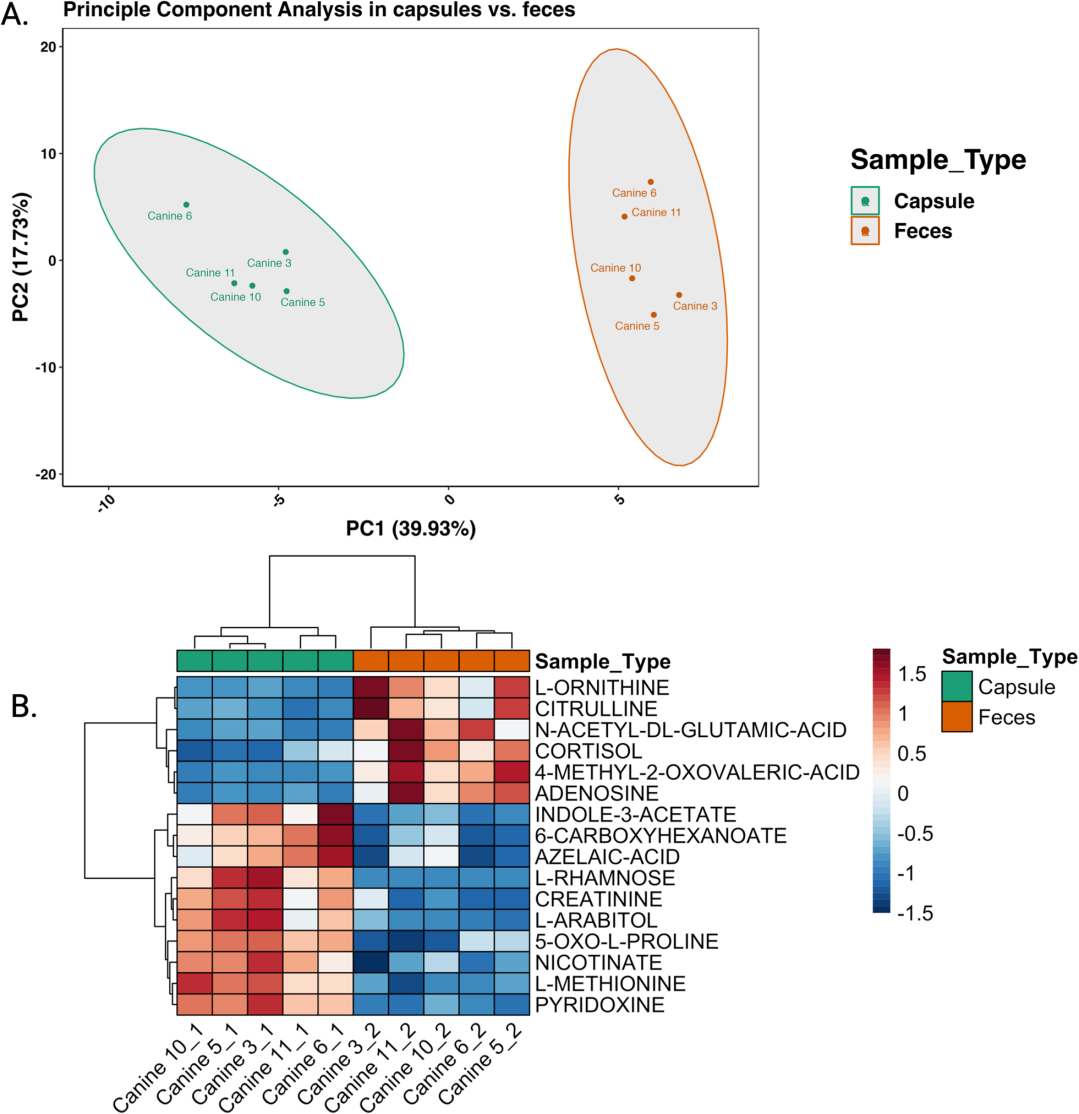
**A.** Principal component analysis of the metabolome showed differences in clustering in the capsule and fecal samples. **B.** As shown in the heatmap, paired *t* test results found 16 metabolites that were significantly different between capsule and fecal samples

Polar fraction metabolites were isolated from 5 out of 6 capsule samples, as one capsule from one dog had a sample volume too low to be included in metabolite extraction and submission for analysis. (Table 1). The corresponding five fecal samples of the dogs were submitted for compared metabolome analysis. Principal component analysis of the metabolome (Fig. 8) showed 2 distinct clusters corresponding to capsule metabolome and fecal metabolome. Heat map analysis showed 16 compounds that were significantly different between the capsule and fecal samples with adjusted p values for multiple comparison using paired t-test ranging between 0.004 and 0.036. Ten metabolites specifically vitamins (nicotinate, pyridoxine), fatty acids (azelaic acid, 6-carboxyhexanoate), amino acids (methionine, 5-oxo-l-proline, creatinine,), sugars (rhamnose, l-arabitol) and indoles (indole-3-acetate) were significantly higher in the capsule than in the feces (Fig. 8B). Additionally, nucleotides (adenosine) amino acids (citrulline, ornithine, n-acetyl-glutamic-acid), keto acids (4-methyl-2-oxovaleric acid) and cortisol, where significantly more prevalent in the feces compared to the capsules (Fig. 8B).

No significant correlation was observed between the recovery of certain metabolites and the abundance of specific bacterial classes in neither capsules nor feces.

### AMR gene (AMRG) recovery

**Fig. 9:**
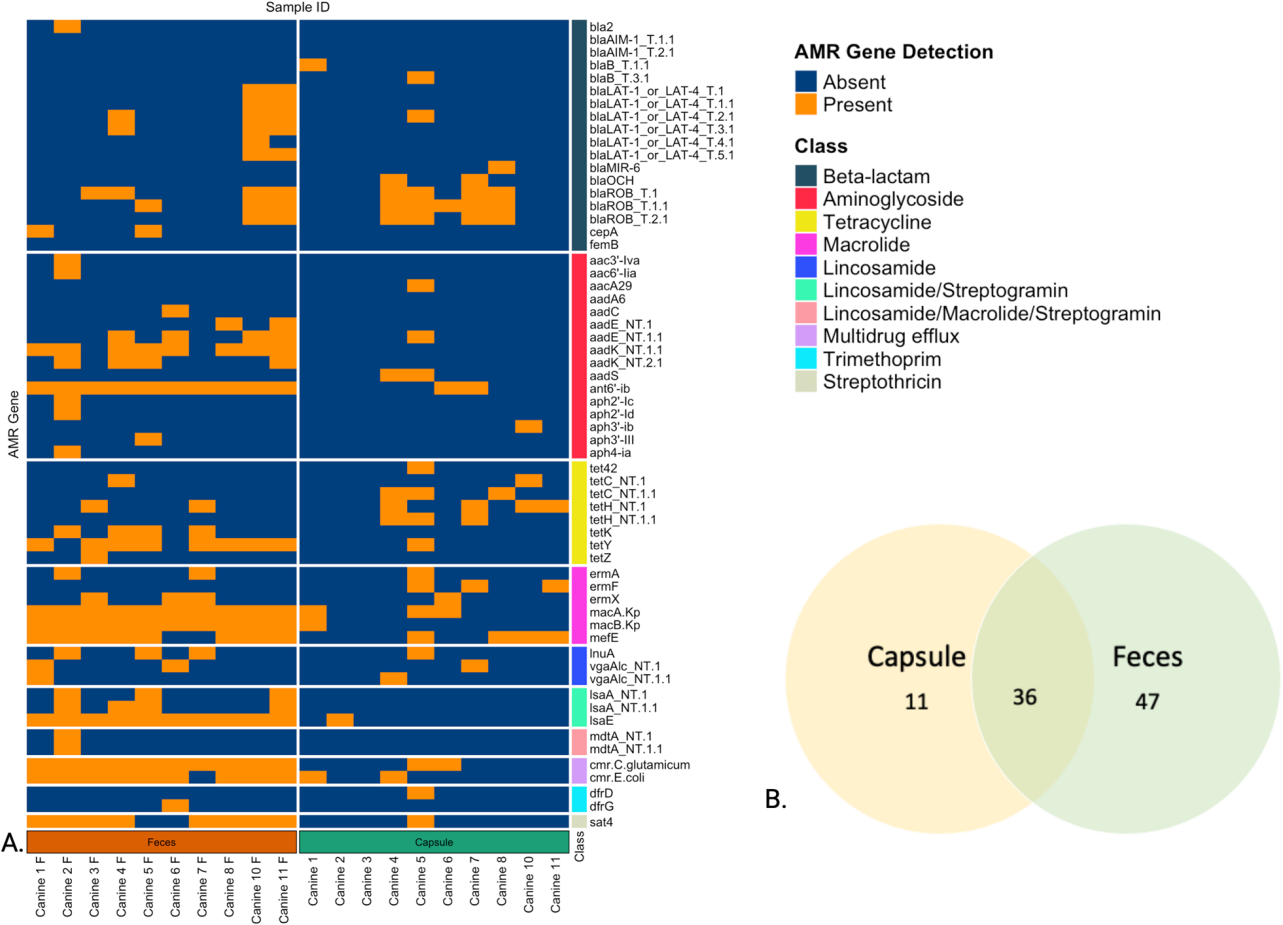
**A** Heatmap distribution of recovery of antimicrobial resistance genes within the capsule and the fecal samples. Each row represents a different AMR gene, and columns correspond to individual samples. Orange indicates the detection of the listed AMR gene, while blue signifies its absence. **B** Venn diagram of sample type where the 74 genes recovered in the samples were found

Of the 796 gene panel, 74 were identified in the samples, 11/74 (14.0.8%) in the capsules alone, 47/74 (63.5%) in the feces alone and 36 /74 (48.6%) in both feces and capsules. The classes of AMRG and distribution of localization of recovery are shown in Fig. 9.

A higher number of resistance genes were recovered in feces compared to the capsule. Specifically, resistance genes to aminoglycosides (*aadK, ant6'-ib, aph2'-Ib, aphA3*), betalactam (*cfxA*_NT.1), macrolide (*ermQ*_NT.1, *macA.Kp_NT.1, macA.Kp_NT.1.1, macB.Kp*), lincosamide (*lsaA*) and tretracycline (*tet40, tet44, tetM, tetW* and *tetY*) were recovered more frequently in feces compared to capsules, as well as genes for multidrug efflux pumps *cmr*.*C.glutamicum* and *cmr*.*E.coli*. (Table 2).

**Table 2:** Antimicrobial resistance genes where recovery within the fecal sample was increased compared to the capsule sample

Antimicrobial class	Region	*P* value Mc Nemar test
Aminoglycoside	aadK_NT.1.1	0.0133
	ant6prime-ib_NT.1	0.0133
	aph2prime-Ib_NT.1	0.0133
	aph2prime-Ib_NT.1.1	0.0412
	aphA3_NT.1	0.0077
Beta lactam	Cfxa_NT.1	0.0233
Multidrug efflux	Cmr.C.glutamicum_NT.1	0.0133
	Cmr.E.coli_NT.1	0.0233
Macrolide	Ermq_NT.1	0.0233
	macA.Kp_NT.1	0.0077
	macA.Kp_NT.1.1	0.0233
	macB.Kp_NT.1	0.0077
Lincosamide/streptogramin	Lsae_NT.1.1	0.0077
Streptothricin	Sat4_NT.1	0.0455
Tetracycline	Tet40_NT.1	0.0412
	Tet44_NT.1	0.0412
	Tetm_NT.1	0.0412
	Tetm_NT.1.1	0.0133
	Tetw_NT.1	0.0412
	Tety_NT.1	0.0233

## Discussion

Noninvasive sampling of the small intestinal microbiome and metabolome, as well as recovery of AMRG in dogs is possible. Mean transit time for the SIMBA™ capsule is within 4 h of previous studies in dogs given engineered capsules, whether telemetric wireless pH sensitive capsules [21] or video endoscope capsules [22, 23]. Administration of the capsule within a meatball was easy for all dogs. One dog (canine 8) chewed the meatballs capsules, and the DNA recovery in one of those capsules was limited (Table 1). Previous studies have administered the engineered capsule alone, either using a pill syringe or manually pilling the dog. Use of a pill syringe or manual administration may decrease the risk of chewing and damage to the capsule but may be more stressful for the dog.

We prioritized DNA extraction and microbiome analysis from the capsules over metabolomic analysis, as this was a non-invasive proof of concept (i.e.: without radiographic follow up of the capsules, or concurrent small intestinal fluid aspirate via endoscopy). A larger number of publications describing the variation of microbiome along the canine gastrointestinal tract are available for comparison with our results, compared to metabolomic description of variation along the GIT. Hence, we prioritized microbiome analysis to allow comparison of our results compared to previously published information. Microbiome analysis showed that there were differences between the capsule and the feces. Alpha diversity measured by the Simpson index and observed diversity indexes was decreased in the capsule. Previous studies showed increase in bacterial diversity along the canine GI tract, with the lowest diversity in the stomach due to gastric acidity and the feces having the higher alpha diversity [27, 28]. Additionally, beta diversity was also significantly different between the capsule and the fecal sample. This is also in accordance with previous studies on changes within the microbiome along the GI tract [5].

The five most abundant phyla in the capsules were *Proteobacteria*, Firmicutes, *Firmicutes A, Bacteroidetes* and *Actinobacteriota*. Previous studies in dogs using either upper GI endoscopic biopsies, or recovery of the small intestinal chyme following euthanasia showed a similar distribution of the microbiota. The jejunum microbiome from previous studies showed a predominance of *Proteobacteria* (46.7%), *Firmicutes* (15.0%), *Actinobacteria* (11.2%), *Spirochaetes* (14.2%), *Bacteroidetes* (6.2%), and *Fusobacteria* (5.4%). These results suggest that capsule sampling occurs within the small intestine, most likely the duodenum [5, 16, 19]. However, in four dogs, relative abundance of phyla within the capsules differed from the other dogs' capsule samples, with specifically, a higher abundance of *Proteobacteria* and *Bacteroidetes* (Fig. 5). This discrepancy in microbiota distribution suggests that the sampling procedure for those samples likely took place in the jejunum [17]. To ascertain the exact location of the opening and of the capsule and sampling of the chyme, dogs could have had serial radiographs as the capsule transited throughout the GIT. The traditional radiographic protocol to follow GI transit following administration of radio-opaque contrast consists in a minimum of 5 radiographs at different time points [29]. Local regulation suggests that dogs should be sedated for the radiographic examination to limit exposure of radiation ions to handlers. Sedation has been shown to decrease GI motility in dogs [30]. As such, to limit possible ionization of these teaching dogs and the staff, unsedated radiographs were only taken if transit time of the capsule exceeded 72 h. One dog had a slower transit time than the others. Radiographs showed the capsule present in the ascending colon. The capsule was excreted 3 h following the diagnostic test. Composition of the fecal microbiome in these dogs was comparable to previously documented, with *Clostridiales, Bacteroides*, and *Fusobacteria* being the most abundant classes [31].

Likewise, there were notable distinctions observed in the metabolome analysis between the metabolites found in the capsule and those present in the feces. The number of metabolomic studies in dogs is growing, but data in this field remains scarce. So far, only one study has investigated the variations in metabolomics within the gastrointestinal tract of healthy dogs [5]. In our investigation, we discovered a higher concentration of water-soluble vitamins in the capsule samples compared to the fecal samples, which aligns with the fact that water-soluble vitamins are absorbed in the small intestine [32]. Furthermore, our study revealed elevated levels of amino acids, sugars, and medium chain fatty acids, in the capsule samples when compared to the feces. This aligns with the findings of Folz et al., who observed higher concentrations of sugars, plant products, di- and tri-peptides in the human proximal small intestine compared to distal samples [26]. Additionally, our results are consistent with previous findings in dogs by Honneffer et al., where creatinine, and methionine were more frequently recovered in the duodenum than in the rectum [5]. However, for many other metabolites, our results differed. Honneffer et al. found higher concentrations in dogs of azelaic acid, indole 3 acetate, and nicotinic acid in feces, with no variation in palmitic acid across the gastrointestinal tract. Similarly, Folz et al. found higher levels of indole 3 acetate in human feces compared to the small intestine. Additionally, both Honneffer and Folz studies reported higher concentrations of nucleosides (thymidine, uridine, adenosine), amino acids (citrulline, ornithine), and plant alkaloids (xanthine and xanthosine) in upper intestinal samples, whereas our findings indicated higher levels in the fecal samples [26]. The clinical significance of these findings remains unknown, and it is possible that diet, as all the dogs in the Honnefer study were fed the same diet, and lifestyle factors contributed to these differences. We have accounted for sex, diet, and housing arrangement in our adjusted values. Given that only one dog fed a hydrolyzed diet had a sample submitted for metabolomic analysis, no specific further analysis were conducted related to effect of diet. Further studies investigating the various metabolites throughout the gastrointestinal tract, the effect of diet and disease processes such as IBD or EPI are warranted.

Lastly, this is to our knowledge, the first time that recovery of resistance genes is attempted in the small intestine. Resistance genes were less frequently recovered in the capsule sample compared to the fecal sample. This may suggest that the resistome is located primarily in the large intestine, although despite normalization there was still a higher level of input DNA for the fecal samples due to half of the capsules having concentrations too low to be detectable by fluorimetry. This novel information is of interest for further investigation, as this may help select specific therapeutic interventions to limit the emergence of resistance genes within the large intestine. Our healthy teaching dogs all had recovery of resistance genes to aminoglycoside, beta lactam, lincosamide, macrolide and tetracycline within their feces. No antimicrobial treatment had been administered in the 3 months prior to the start of the study. These findings of carriage of antimicrobial resistance are like previously published studies in healthy dogs presenting to dog shows or veterinary practices for wellness examination [33–35].

To ascertain the exact location of the sampling of the SI is unknown and whether both capsules in the same dog sampled the SI at the same location, Radiographic follow up of the capsule may be considered. However, as mentioned above, radiographic evaluation of canine gastrointestinal transit requires numerous serial studies, possible sedation which impacts gastrointestinal transit time and negates the non-invasive approach of the technology. A recent study in humans using a similar technology showed recovery of a sufficient amount of intestinal chyme within 1 capsule to allow microbiome, proteomic and metabolomic analysis using a similar size capsule. Improvement in the current technology could allow similar sampling [26]. Our study had a few limitations. First, the microbiome and metabolomic compositions could possibly have been altered during gastrointestinal transit in the sampled capsule, as biological processes may have persisted. To prevent this, the SIMBA capsule contains a bactericidal quencher which halts bacterial growth and metabolism. A recent human study using the SIMBA capsule demonstrated consistent findings between capsule multi-omic analysis and contemporaneous small intestinal endoscopic aspirates sample [36]. Moreover, we only performed a negative scan for metabolomic analysis which likely decreased the breadth of metabolite coverage. Groves et al. demonstrated that 80% of detectable metabolites of interest were detected using a negative scan [37]. Agmatine, gamma-amino butyrate, tyramine are some of the compounds which could have been detected by the positive mode if performed. Given the proof-of-concept type of study, the primary objective was to optimize sample throughput rather than focusing on expanding the number of metabolites detected. Lastly, the quantity of DNA recovered from the capsule was small compared to the quantity available with the feces. Additional investigations are necessary to comprehensively elucidate the influence of the limited amount of DNA on the precision of PCR AMR isolation and diagnostic assays employed in our research.

## Conclusions

Noninvasive sampling of the small animal microbiome and metabolome in dogs is feasible with minimal adverse effects. Use of an engineered capsule allowed for microbiome and metabolome analysis and showed significant differences compared to fecal samples. In addition, capsule samples also allowed for identification of AMRG. Further studies should be performed to test the reliability and safety of the capsule sampling in sick dogs.

## Methods

### This was a prospective proof of concept study

#### Animals

This study was approved by the University of Calgary Animal Care Committee (ACC21-172). Eleven beagle dogs, part of a teaching colony, were prospectively enrolled. Dogs were deemed healthy based on clinical examination and routine veterinary care provided. No teaching requirements were performed during the week prior, week of and the week following the intervention. To be included in the study, dogs had to weigh more than 8 kgs, have no history of GI disease or GI surgery and not be receiving any prokinetic drugs which may interfere with transit time. Dogs were housed in individual kennels at night but spent time together in small groups of 3 during the day, with 3 to 5 h of canine social contact indoor and outdoor per day and 1 to 5 h of human social time per day. In addition, during the duration of the study, the dogs had 20–30 min leash walks once to twice per day. The dogs are fed twice daily at 7:30 am and 3:30 p m a canine commercial diet (Table 1) (7 study dogs received Royal Canin Dental, 2 dogs Royal Canin Hypoallergenic Hydrolyzed Protein, one dog each Royal Canin Satiety and 1 Purina Veterinary Diets Essential Care–Dry + Science Diet Beef & Barley Entree – Canned). Between 7 pm and 7am, indoor lights are turned off.

#### Administration protocol

**Fig. 10:**
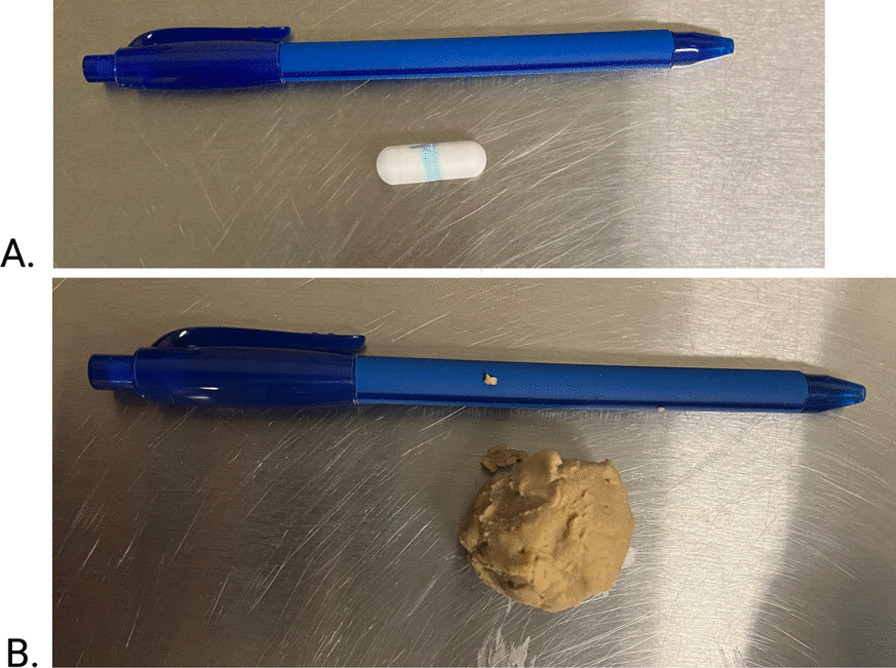
**A.** Engineered Small Intestinal MicroBiome Aspiration (SIMBA™) capsule (Nimble Sciences) with a ball pen for size comparison. **B.** SIMBA™ capsule placed in a meatball of can food (Royal Canin Veterinary®, hypoallergenic hydrolyzed protein loaf, Mars inc. Puslinch, Ontario)

**Fig. 11:**
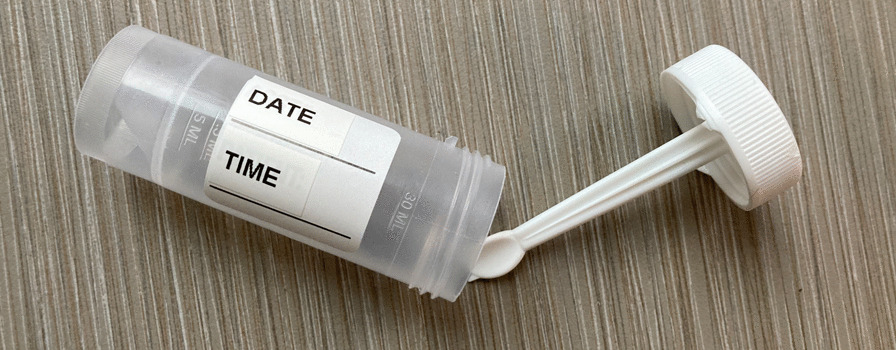
Fecal collection and storage tube

Dogs were administered a total of 2 capsules each which were engraved serial numbers to allow matching of dogs and capsule and fecal samples. Each capsule was placed in a cherry size meatball of can food (Royal Canin Veterinary®, hypoallergenic hydrolyzed protein loaf, Mars inc. Puslinch, Ontario) to facilitate administration (Fig. 10). The day prior, dogs were fed their normal diet following routine schedule. The morning of the study, capsules were administered via feeding 2 small meatballs (Additional file 1: Video S1). To allow sufficient time for capsules to transit through the stomach, the morning meal (consisting of their regular diet (Table 1)) was delayed until 2 h post meatball ingestion. For the remainder of the study time (until all capsules were accounted for), dogs ate their diet with routine feeding schedule and activities. Time at which the capsules were administered was recorded as t0. Starting at 8 h post capsule administration, feces of each dog was collected and searched for presence of the capsule(s) for up to 96 h. Feces were collected and searched every two hours from 7:30 am to 3:30 pm and once between 8:30 and 9:00 pm. No fecal collection was performed overnight between 9:00 pm and 7:30 am the following morning. Each capsule was manually retrieved and stored in a clean fecal collection tube (Fig. 11), with at least 2 g of feces. Once collected, capsule and fecal samples were kept at 4 °C submitted to Nimble Science for sample processing. A rescue protocol was planned in case of slow GI transit time or concerns of gastrointestinal obstruction from the capsule. If at 72 h post capsule ingestion, capsules were not yet retrieved from feces, unsedated abdominal radiographs were performed in order to determine whether the radio-opaque capsule was seen within the GI tract. If at any time point, dogs showed clinical signs suggestive of GI obstruction (decreased energy and/or appetite, vomiting) physical examination, bloodwork and 3 view abdominal radiographs would have been performed to look for evidence of GI obstruction.

#### Capsule and fecal samples

The capsules used are a standard 00 size, measuring 23.4 mm long × 8.6 mm wide. The capsule has a shell with a gastric-resistant coating, which dissolves passively from the intestinal chyme once the coating is exposed in a neutral pH. Then, the capsule collects an average of 80 μL of intestinal fluid from multiple large open ports which are autonomously sealed by a time-controlled dissolvable latch. A quencher embedded in the capsule is released to quench the growth of the microbes in the collected sample [36].

A fecal sample of at least 2 g, is collected from a portion of the stool immediately adjacent to the capsule. That sample is paired with each capsule or pair of capsules retrieved from a dog. The fecal and capsule samples collected from one dog at the same time were stored in the same fecal collection tube and stored at 4 °C prior to and during delivery to Nimble Science processing facility. Once received, the feces and capsules were separated so that the capsules could be immediately processed for sample removal. Feces were mechanically homogenized using the spoon attached to the fecal collection tube, placed in a styrofoam box packed with dry ice that was closed and placed in a − 80 °C freezer. These samples remained frozen until analysis. Out of the two capsule samples collected, the heavier sample out of two were first selected for DNA extraction. DNA analysis was prioritized over metabolomic analysis and if needed, the second capsule was used for DNA analysis instead of metabolomic analysis. The Qiagen QIAamp PowerFecal Pro DNA Kit, which was used to extract DNA from capsule and fecal samples, uses a novel bead tube in combination with chemicals for efficient mechanical and chemical lysis. The kit also uses proprietary Inhibitor Removal Technology to eliminate common inhibitors found in stool and gut samples. DNA extracted from samples and feces using this kit were split into two aliquots; microbiome 16S rRNA analysis was performed on one aliquot and PCR for detection of antimicrobial resistance genes (AMRG) was performed on the second aliquot. Of the 22 paired capsule samples retrieved from each of 11 dogs, a total of 14 were assigned to DNA extraction for downstream 16S rRNA and AMRG PCR, while the remaining 6 capsule samples were designated to polar-fraction metabolite extraction for downstream untargeted metabolomics analysis. The capsule samples with heavier sample weight between the pair were used for DNA extraction, except for in cases of process-related complications such as sample loss or potential contamination, where the lighter capsule also underwent DNA extraction.

### DNA extraction

DNA extraction was performed with the Qiagen QIAamp PowerFecal Pro DNA Kit, following the manufacturer’s protocol with minor modifications regarding bead beating method and final elution volume. Sequencing analysis was performed using the Illumina MiSeq System.

The 16S rRNA gene V4 variable region was amplified using the 515F and 806R for the V4 region primers in a 35 cycle PCR using the KAPA HiFi HotStart master mix (Roche Sequencing). A maximum volume of 13.5 µl of template DNA was used for all capsule samples, while 2.5 µl of template DNA was used for fecal samples. The conditions for the thermocycler were as follows: 98 °C for 2 min, followed by 35 cycles of 98 °C for 30 s, 55 °C for 30 s and 72 °C for 20 s, after which a final elongation step at 72 °C for 7 min. Amplified PCR products were checked in a 1% agarose gel. The PCR products were then purified using NucleoMag NGS Clean-up and Size Select (Macherey–Nagel), concentrations normalized using SequalPrep Normalization Plate (Invitrogen), and pooled. DNA quality of the pooled products was analyzed by TapeStation (Agilent). The pooled library was then denatured and prepared for loading on an Illumina MiSeq cartridge with a 5% PhiX Control.

### Microbiome analysis

Paired-end sequencing was executed, yielding reads of 250 base pairs in length. The resulting sequencing data underwent de-multiplexing and conversion to Fastq format via Illumina’s bcl2fastq software [38]. Primers were not sequenced, so Cutadapt v1.16 was employed for initial quality trimming with minimum quality score of 20 [39]. Subsequent analyses were conducted in R version 4.0.2, utilizing the DADA2 pipeline (v 1.16.0) as per the recommended guidelines [40]. Forward and reverse reads were cleaned to eliminate Phix reads and were filtered based on parameters maxEE = c(2,2) and truncQ = 2. The denoising step was followed by merging reads, requiring a minimum overlap of 12 base pairs, and allowing no mismatches. Chimeric sequences were identified and removed using consensus method to generate an amplicon sequence variant (ASV) table. Classification and taxonomy assignment was done using RDP classifier (a naive Bayesian classifier) [41] and GTDB v4.2 database [42].

The bacterial composition and phylogeny information were used to identify outliers and remove if there were capsules that had collected samples within the colon. Negative control samples were used to find and remove possible contaminations in samples using the Decontam package (method = prevalence, threshold = 0.2) [43]. For amplicon sequencing, capsules yielding a greater sample mass were selected. Additionally, any samples yielding fewer than 2000 sequencing reads were excluded from further study. ASV and taxonomy filtration was done by removing ASVs not assigned to any known kingdom or phylum. Rare phyla characterized by a sum prevalence and sum abundance of less than 10 and 250, respectively, were removed to focus on more significant microbial signatures. Finally, the filtration process was refined to include only those amplicon sequence variants (ASVs) that demonstrated a relative abundance exceeding 0.005%, as well as a prevalence over 10%. These filtration cleaning steps lead to a total abundance of 1,812,610 taxa across 20 samples.

### Metabolome analysis

The metabolite extraction was performed using a methanol and acetonitrile-based protocol adopted from previously published studies [44–46] for the isolation of polar metabolites for downstream untargeted HILIC Liquid Chromatography–Mass Spectrometry analysis. A minimum volume of 40 ul of the sample is required to run untargeted HILIC LC–MS analysis. For capsule samples, this volume represents an absolute minimum of a D15 dilution, prepared during metabolite extraction using a methanol and acetonitrile-based protocol and of which 100 µl was submitted for analysis. For fecal samples, a D5 dilution was prepared during metabolite extraction using a methanol protocol and of which 100 ul was submitted for analysis. Fecal samples were further diluted to match the final dilution of capsule samples used for analysis. Untargeted HILIC LC–MS analysis was performed using the Thermo Scientific Q Exactive Orbitrap LC–MS/MS System coupled to a Vanquish™ UHPLC System (Thermo-Fisher). Chromatographical separation of metabolites was performed on Syncronis HILIC UHPLC column (2.1 mm × 100 mm × 1.7um, Thermo-Fisher) at the flow rate of 600 uL/min using a binary solvent system: solvent A, 20 mM ammonium formate pH 3.0 in mass spectrometry grade H_2_0 and solvent B, mass spectrometry grade acetonitrile with 0.1% formic acid (%v/v). The following gradient was used: 0–2 min, 100%B; 2–7 min, 100–80%B; 7–10 min, 80–5%B; 10–12 min, 5% B; 12–13 min, 5–100%B; 13–15 min, 100%B. Sample injection volume was 2 uL. The mass spectrometer was run in negative full scan mode at a resolution of 240,000 scanning from 50 to 750 m/z. Metabolite data was analyzed by El-MAVEN software package [47, 48]. Metabolites were identified by matching observed m/z signals (± 10 ppm) and chromatographic retention times to those observed from commercial metabolite standards (LMSLS™ Sigma-Aldrich). Next, metabolites were quantified by comparison to an eight-point quantification curve of metabolite standards.

### AMR gene analysis

Aliquots of 20 uL DNA were shipped on dry ice for this analysis. DNA concentrations were normalized to ≤ 4 ng/ul, and the two primer pools for the AmpliSeq for Illumina AMR Community Panel were used to amplify up to 815 amplicons covering 478 AMR genes in 28 different classes. In addition to the 26 samples from dogs (14 capsules and 12 fecals), 5 negative controls in total were included to control for each stage of processing. The Ampliseq Library Plus kit was then used to make sequencing libraries with unique dual indices added to each sample according to the manufacturer’s instructions. Sequencing was performed on the MiSeq platform with 2 × 150 bp chemistry, with all 32 libraries pooled on one v3 cartridge. Final analysis of the AMRG was conducted on the 10 paired capsule and fecal samples.

### Statistical methods

Sample size calculation: No previous data was available for sample size calculation. Using the resource equation method (n = DF/*k* + 1), where n is the number of subjects, DF is the between-subject error and *k* is number of groups, a sample size of 11 was judged to be sufficient [49].

Normality using the Shapiro Wilk test test and descriptive statistics were calculated as appropriate. Analysis was performed on the corresponding paired capsule and fecal samples. Correlation between the mean transit time and mean capsule sample weight was calculated with Spearman’s rank correlation. Outliers were identified using Grubb’s tests [50]. A paired *t* test was used to compare mean capsule DNA weight and mean fecal DNA weight. Capsules mean DNA weighing < 0.0100 ng/ul concentration (listed as OOR in Table 1) and their paired fecal samples were excluded from the analysis. Alpha diversity was measured by calculating the Simpson, Shannon and observed indexes. Statistical analyses were conducted on two groups of samples using the paired *t*-test and the Wilcoxon signed-rank exact test for data with normal distribution and without normal distribution, respectively. For beta diversity analysis, the data were log-transformed, and the results were visualized using principal coordinate analysis (PCoA) based on Bray–Curtis, Weighted Unifrac, and Unweighted Unifrac dissimilarity metrics. Changes in the bacterial community composition were statistically assessed using permutational multivariate analysis of variance (PERMANOVA) with 999 permutations, considering the paired nature of the samples. ANOSIM with 999 permutations was also performed to quantitatively compare the bacterial community differences between paired capsule and fecal samples. *P* values were corrected using Benjamin Hochberg method and results were considered statistically significant with *P* ≤ 0.05. Top 5 phyla to genera were also identified in each sample type and was statistically compared between capsule and fecal samples using the Wilcoxon signed rank test. Taxa bar plots of relative abundance data were also generated at phyla to family taxonomy levels. We also conducted an ASV-level analysis to visualize the overlap and unique ASVs in capsule versus fecal samples using a Venn diagram. The raw metabolomics data underwent a series of preprocessing steps including median normalization per sample, logarithmic transformation, and scaling for metabolites. To visualize the distribution of metabolites and assess patterns within the dataset, principal component analysis (PCA) was carried out after dimensional reduction by mean-centering and scaling the data. Statistical analysis employed paired *t*-tests with Benjamin-Hochberg correction (*P* ≤ 0.05) to identify significant metabolite differences between sample types. The results of the analysis were presented using a heatmap, which was generated using the Ward.D2 hierarchical clustering method and the Euclidean distance metric.

Final analysis of the AMRG was conducted on the 10 paired capsule and fecal samples. R version 4.3.0 [51] and the following R packages were used to conduct the corresponding analysis: BiocManager v. 1.30.22 [52], circlize v. 0.4.15 [53], ComplexHeatmap v. 2.16.0 [54, 55], devtools v. 2.4.5 [56], knitr v. 1.45 [57–59], Polychrome v. 1.5.1  [60], rmarkdown v. 2.25 [61–63], and tidyverse v. 2.0.0  [64]. The frequency of detection of AMR genes between capsule and feces detected in more than 1 sample was compared using the McNemar test. A *P* value less than 0.05 was considered statistically significant in all tests. All analyses will be performed using commercial software R version 4.3.0 [51].

### Supplementary Information

**Additional file 1:** Administration of meatballs containing one SIMBA capsule each.

## Data Availability

Available at NCBI SRA under BioProject PRJNA1000616.

## Abbreviations

CI: Confidence interval
EPI: Exocrine pancreatitic insufficency
GI: Gastrointestinal
GIT: Gastrointestinal tract
IBD: Inflammatory bowel disease
IQR: Interquartile range
SI: Small intestine
Std: Standard deviation
